# Supplementary material for: Single-cell transcriptome analysis of embryonic and adult endothelial cells allows to rank the hemogenic potential of post-natal endothelium
Source: Sci Rep. 2022 Jul 16;12:12177. doi: 10.1038/s41598-022-16127-0 (PMC9288434; doi:10.1038/s41598-022-16127-0)
Supplement: Supplementary file 1 — Supplementary Information 1. [file 41598_2022_16127_MOESM1_ESM.pdf]

Supplemental Information

Single-cell transcriptome analysis of embryonic and adult endothelial cells allows to rank the hemogenic potential of post-natal endothelium

Artem Adamov, Yasmin Natalia Serina Secanechia, and Christophe Lancrin

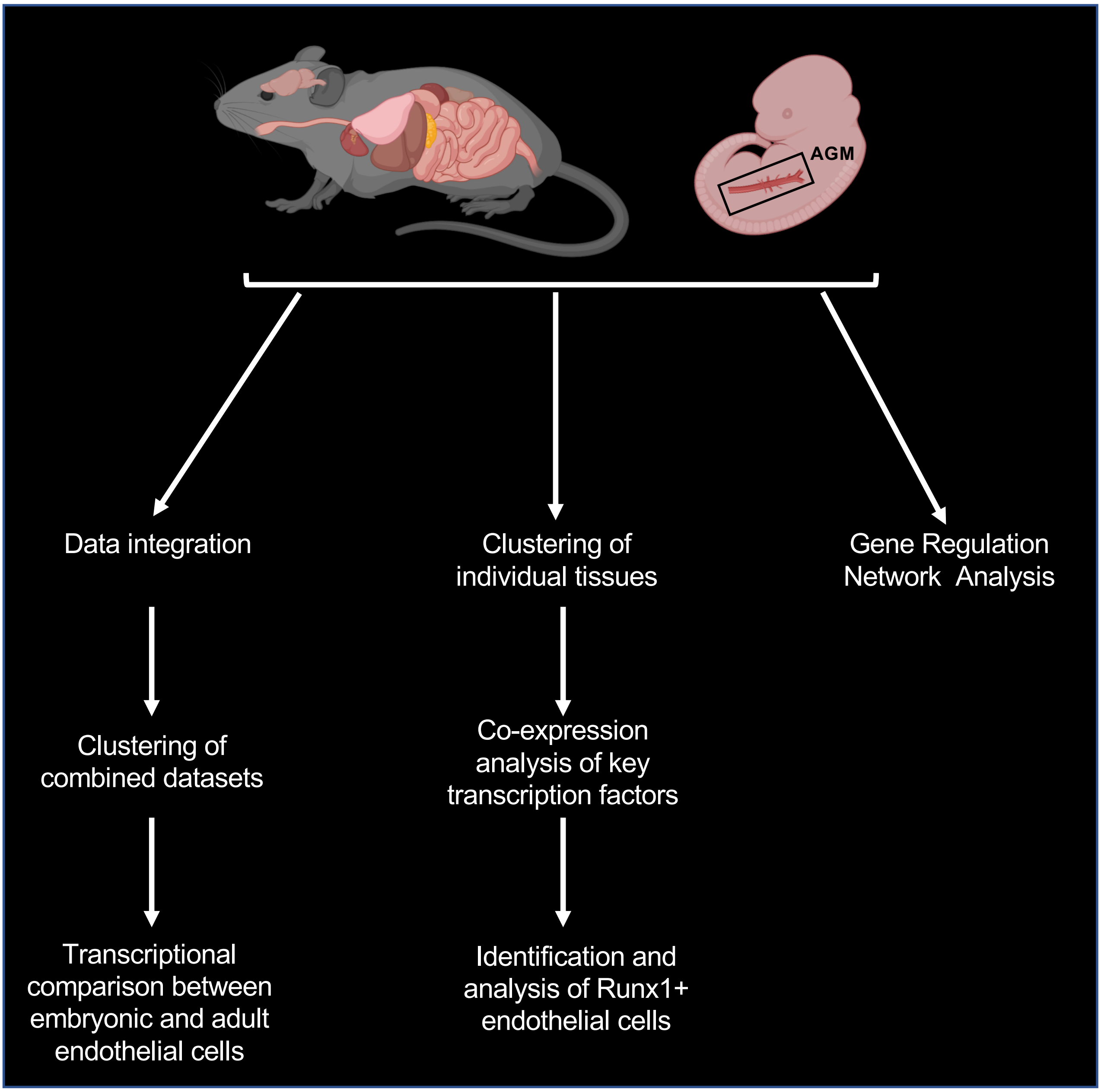
AGM
Data integration
Clustering of individual tissues
Gene Regulation Network Analysis
Clustering of combined datasets
Co-expression analysis of key transcription factors
Transcriptional comparison between embryonic and adult endothelial cells
Identification and analysis of Runx1+ endothelial cells

Supplementary Fig.1: Comparative analysis of murine embryonic and adult endothelial cells. Figure was made in BioRender - biorender.com.

a

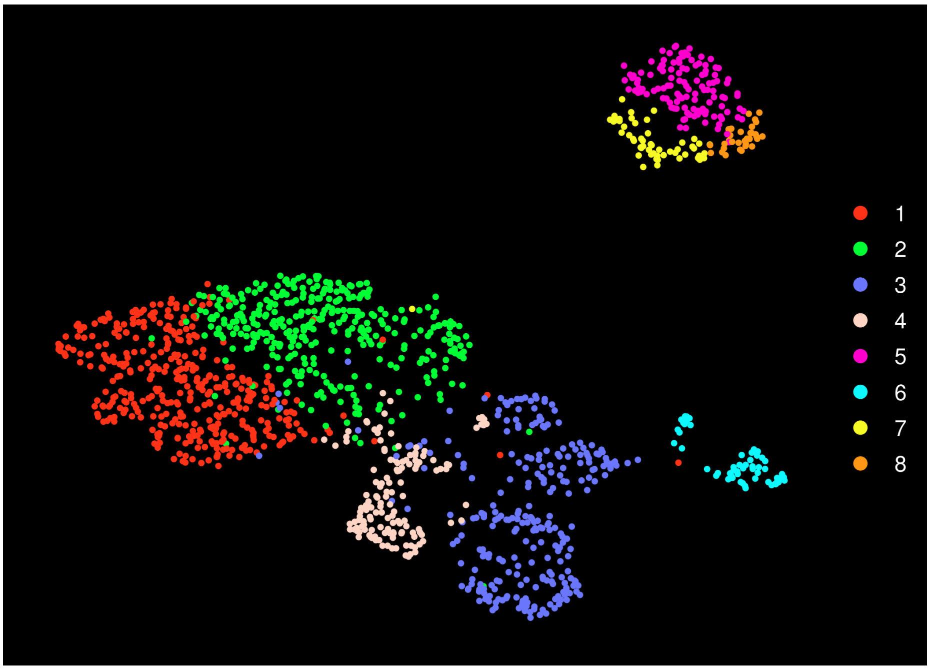
1
2
3
4
5
6
7
8

b

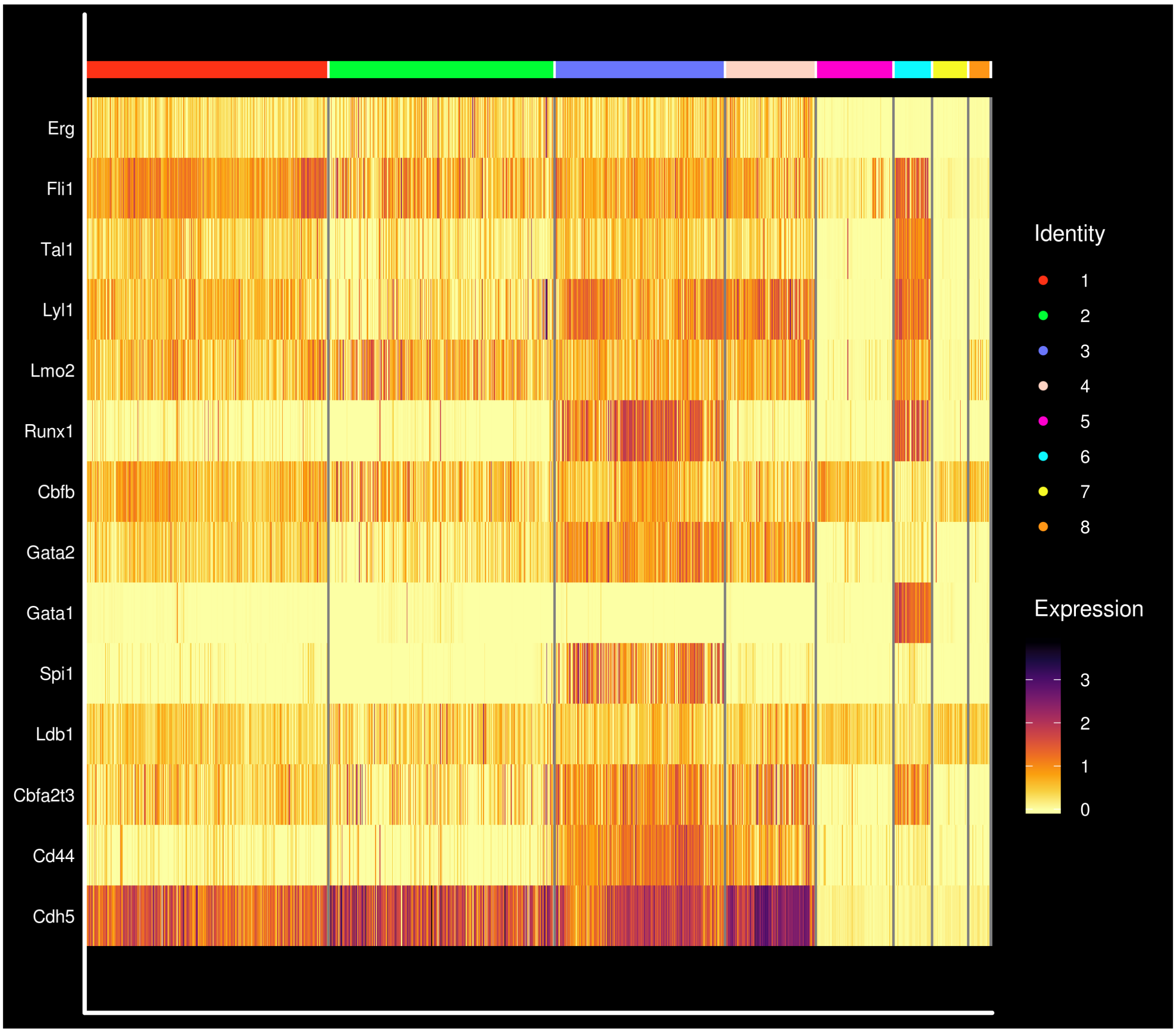
Erg
Fli1
Tal1
Lyl1
Lmo2
Runx1
Cbfb
Gata2
Gata1
Spi1
Ldb1
Cbfa2t3
Cd44
Cdh5
Identity
1
2
3
4
5
6
7
8
Expression
3
2
1
0

Supplementary Fig. 2: Clustering analysis of Embryo_dataset_1. a) UMAP plots showing the clustering analysis result; **b)** Gene expression heatmap for a selection of key genes involved in the EHT process.

Supplementary Fig. 3

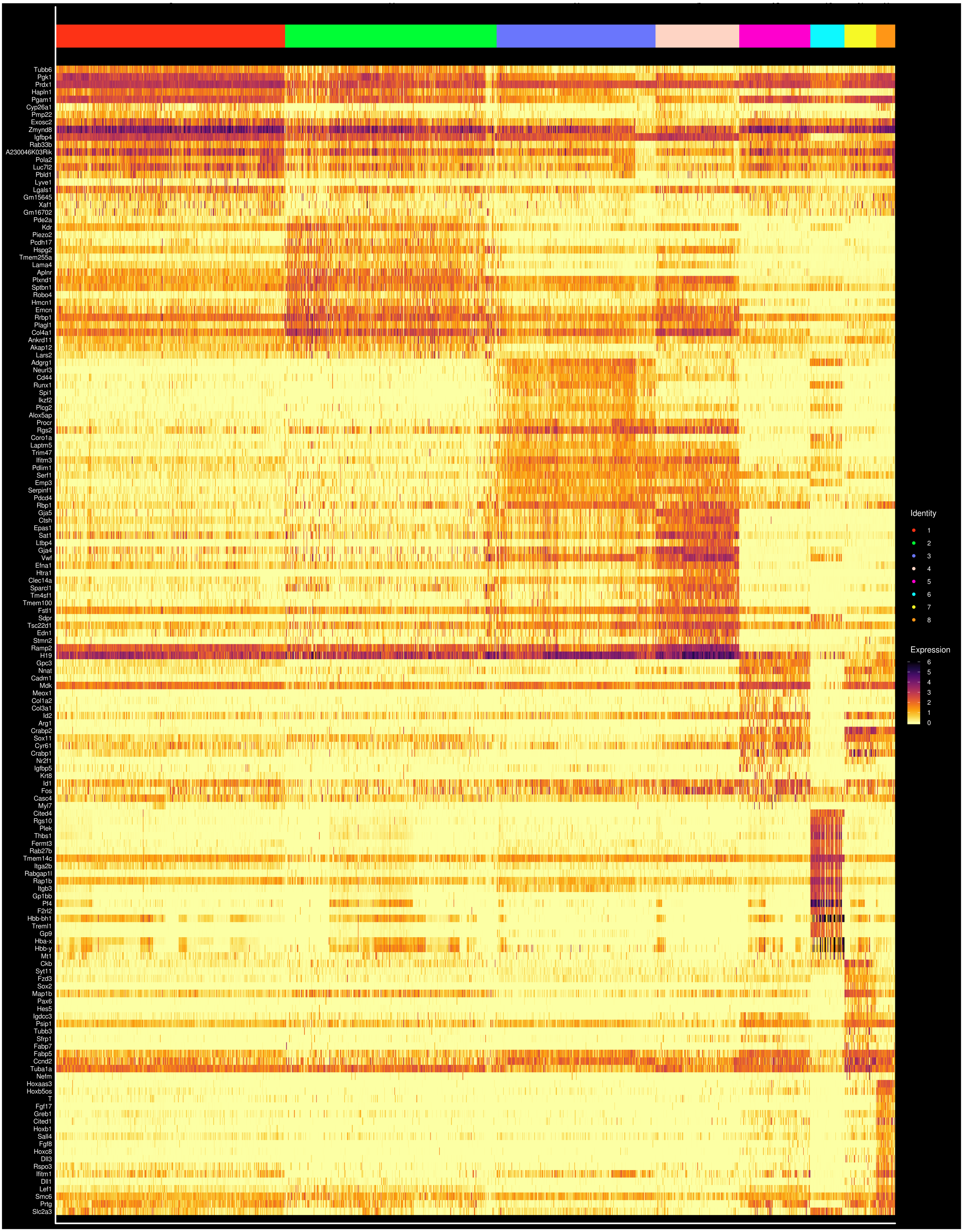

Supplementary Fig. 3: Marker genes of Embryo_dataset_1 clusters. Expression heatmap for marker genes for the eight indicated clusters.

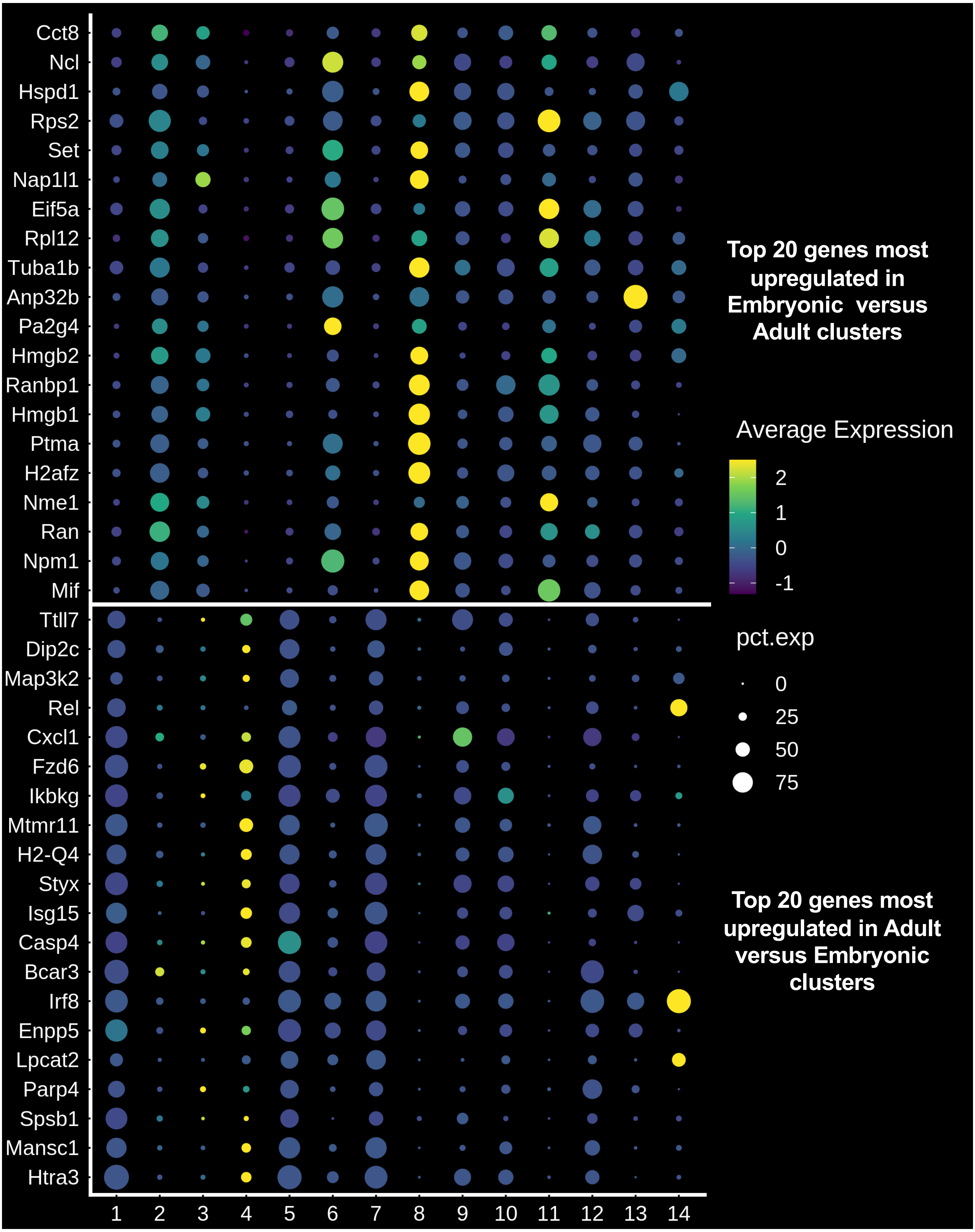

Cell Clusters

Supplementary Fig. 4: Differential gene expression analysis between the embryonic and adult derived cell clusters from Figure 2. Clusters 1, 5, 7, 10, 12, 13 were put together in the Adult group. On the other hand, Clusters 2, 3, 6, 8, 11 and 14 were affiliated to the Embryonic group. A differential expression test of Embryonic vs Adult group was performed. Dot plots indicating the expression and percentage of expression of the genes listed in the graph.

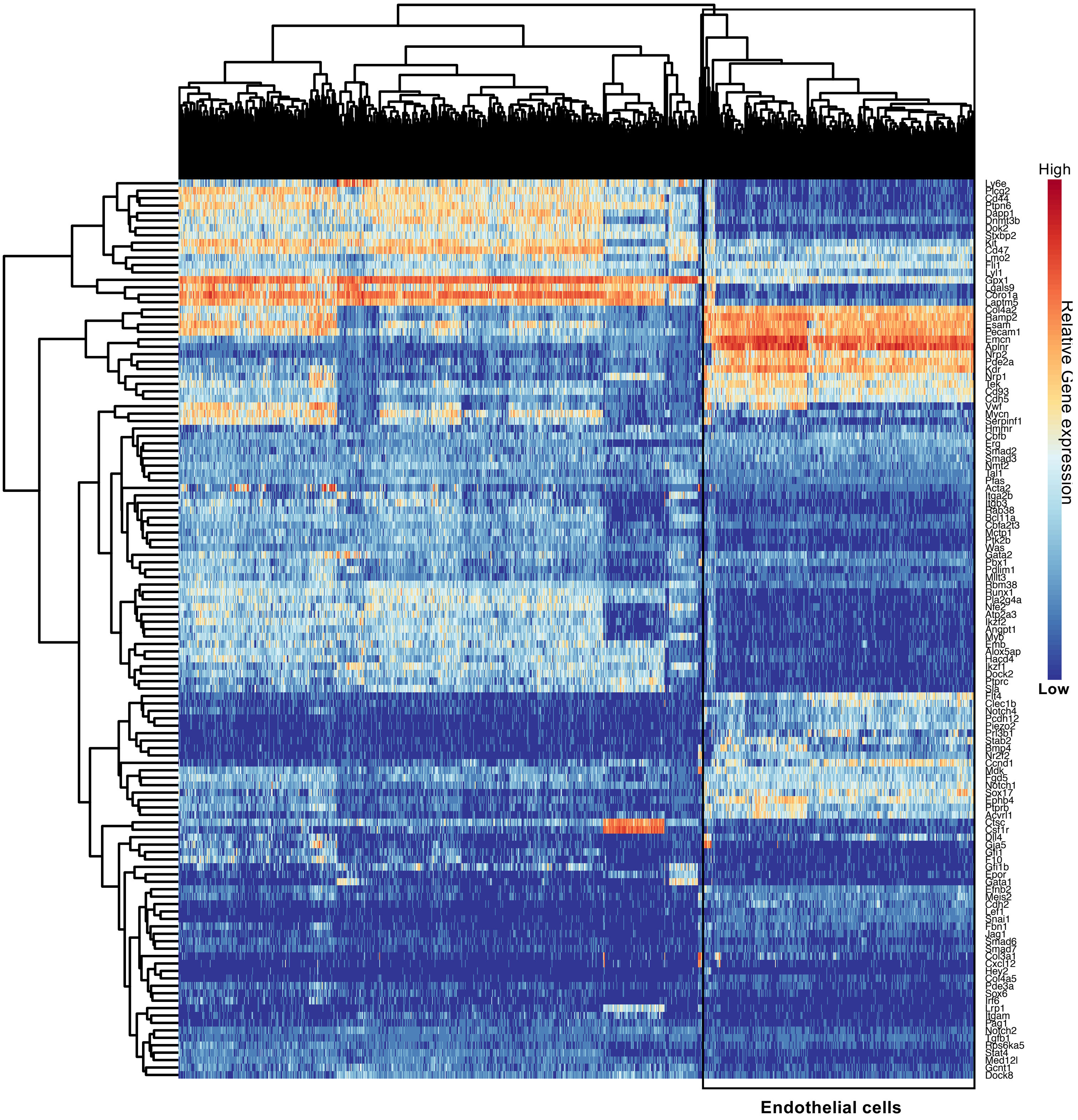
High
Relative Gene expression
Low
Ly6e
Plcg2
Cd44
Ptpn6
Dapp1
Dnmt3b
Dok2
Stxbp2
Kit
Cd47
Lmo2
Fli1
Lyl1
Gpx1
Lgals9
Coro1a
Laptm5
Col4a2
Ramp2
Esam
Pecam1
Emcn
Aplnr
Nrp2
Pde2a
Kdr
Nrp1
Tek
Cd93
Cdh5
Vwf
Mycn
Serpinf1
Hmmr
Cbfb
Erg
Smad2
Smad3
Nmt2
Tal1
Pfas
Acta2
Itga2b
Itgb3
Rab38
Bcl11a
Cbfa2t3
Mctp1
Ptk2b
Was
Gata2
Pbx1
Pdlim1
Mllt3
Rbm38
Runx1
Pla2g4a
Nfe2
Atp2a3
Ikzf2
Angpt1
Myb
Emb
Alox5ap
Hacd4
Ikzf1
Dock2
Ptprc
Sla
Flt4
Clec1b
Notch4
Pcdh12
Piezo2
Prl3b1
Stab2
Bmp4
Nr2f2
Ccnd1
Mdk
Fgd5
Notch1
Sox17
Ephb4
Ptprb
Acvrl1
Ctsc
Csf1r
Dll4
Gja5
Gfi1
F10
Gfi1b
Epor
Gata1
Efnb2
Meis2
Cdh2
Lef1
Snai1
Fbn1
Jag1
Smad6
Smad7
Col3a1
Cxcl12
Hey2
Col4a5
Pde3a
Sox6
Irf6
Lrp1
Itgam
Pag1
Notch2
Tgfb1
Rps6ka5
Stat4
Med12l
Gcnt1
Dock8
Endothelial cells

Supplementary Fig. 5: Clustering analysis of cells from Embryo_dataset_2. Heatmap plot of gene expression in the cells from the Embryo_dataset_2 using a gene list defined in Oatley et al. (2020). The endothelial cells are highlighted. Color scale bar indicates relative gene expression.

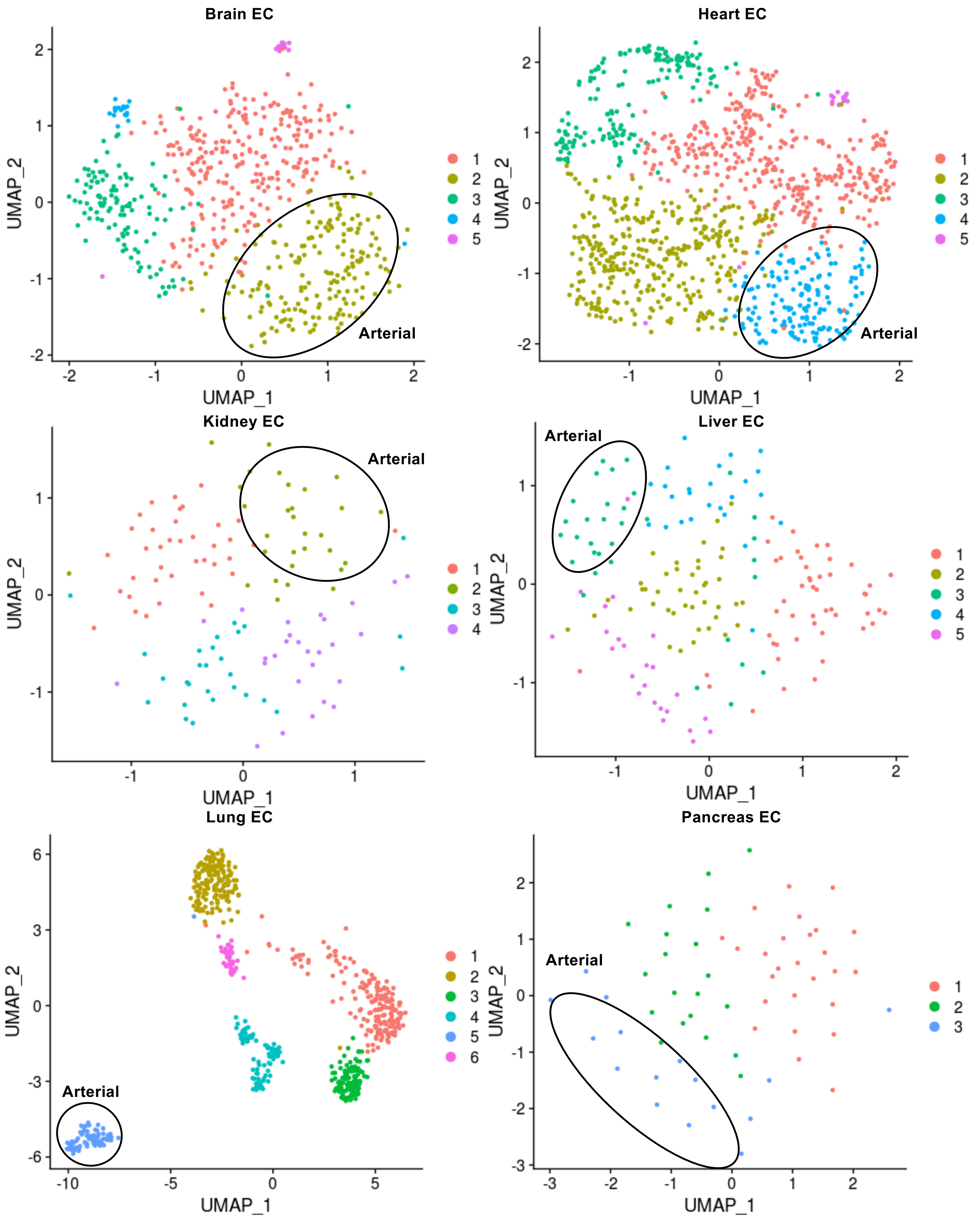
Brain EC
Arterial
Heart EC
Arterial
Kidney EC
Arterial
Liver EC
Arterial
Lung EC
Arterial
Pancreas EC
Arterial
UMAP_1
UMAP_2

Supplementary Fig. 6: Clustering analysis of endothelial cells from Tabula Muris. Umap plots of the results of the endothelial cell clustering analysis of the six indicated tissues. Clusters expressing arterial markers are highlighted with an ellipse.

Brain EC

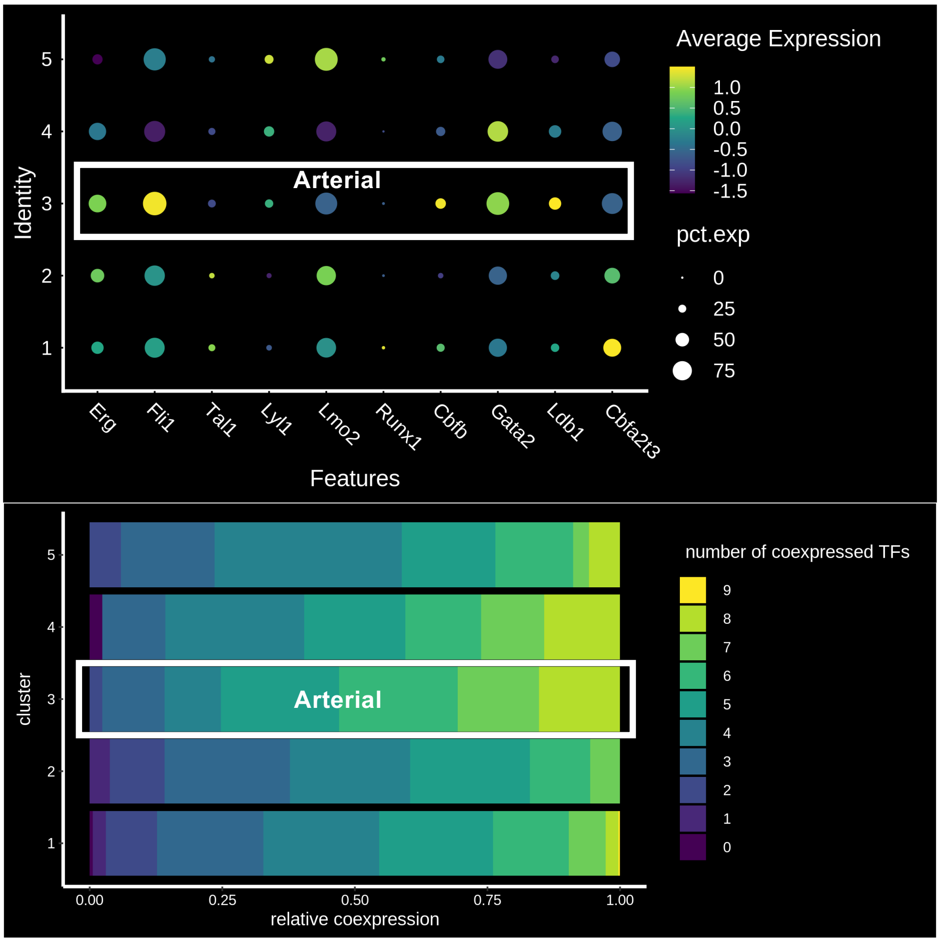

Heart EC

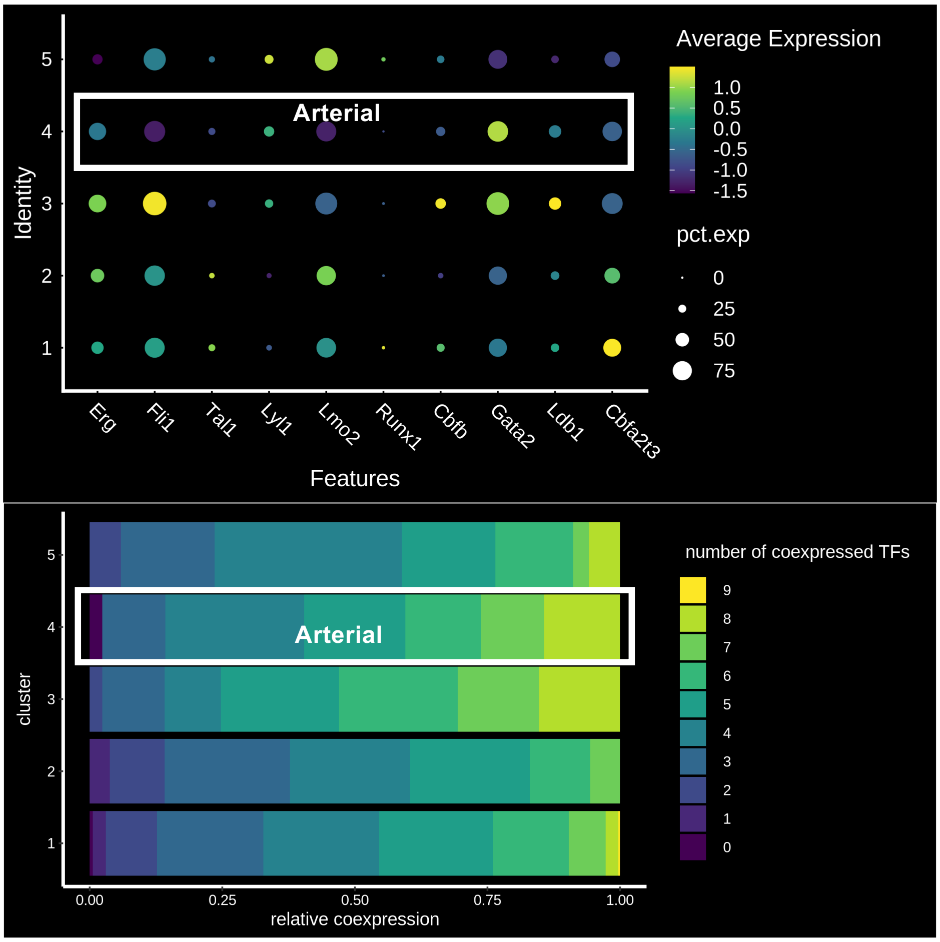

Supplementary Fig. 7

Kidney EC

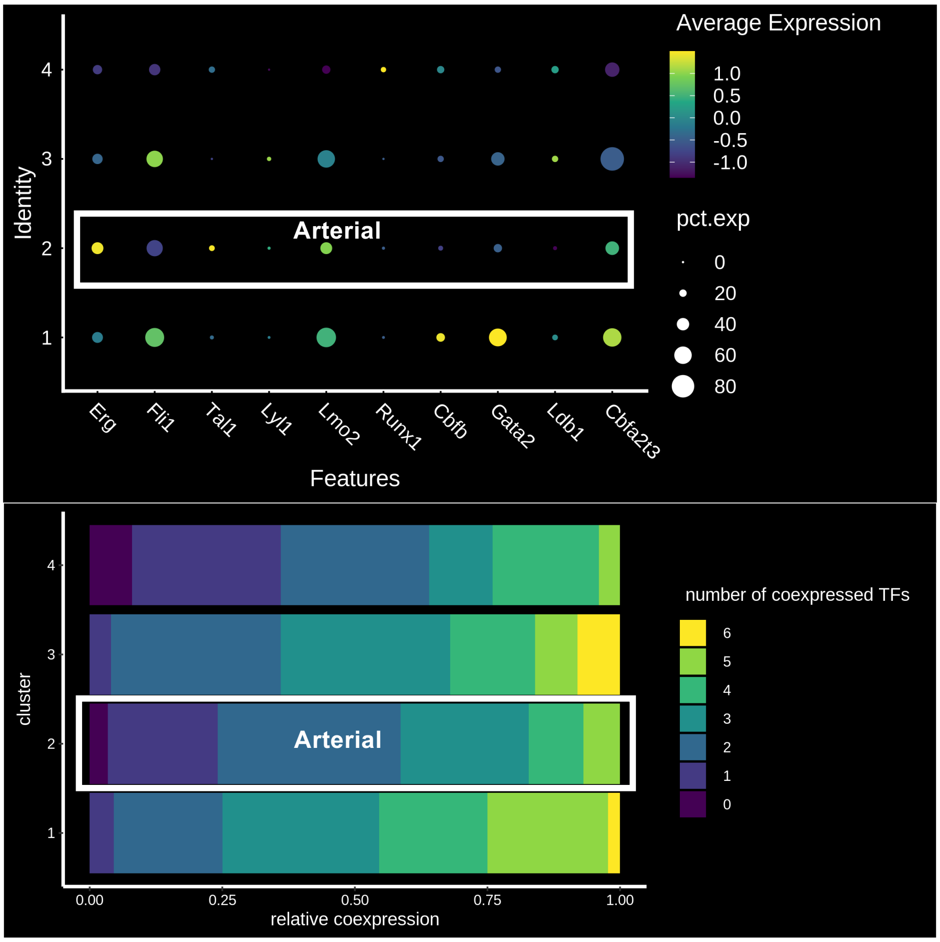

Liver EC

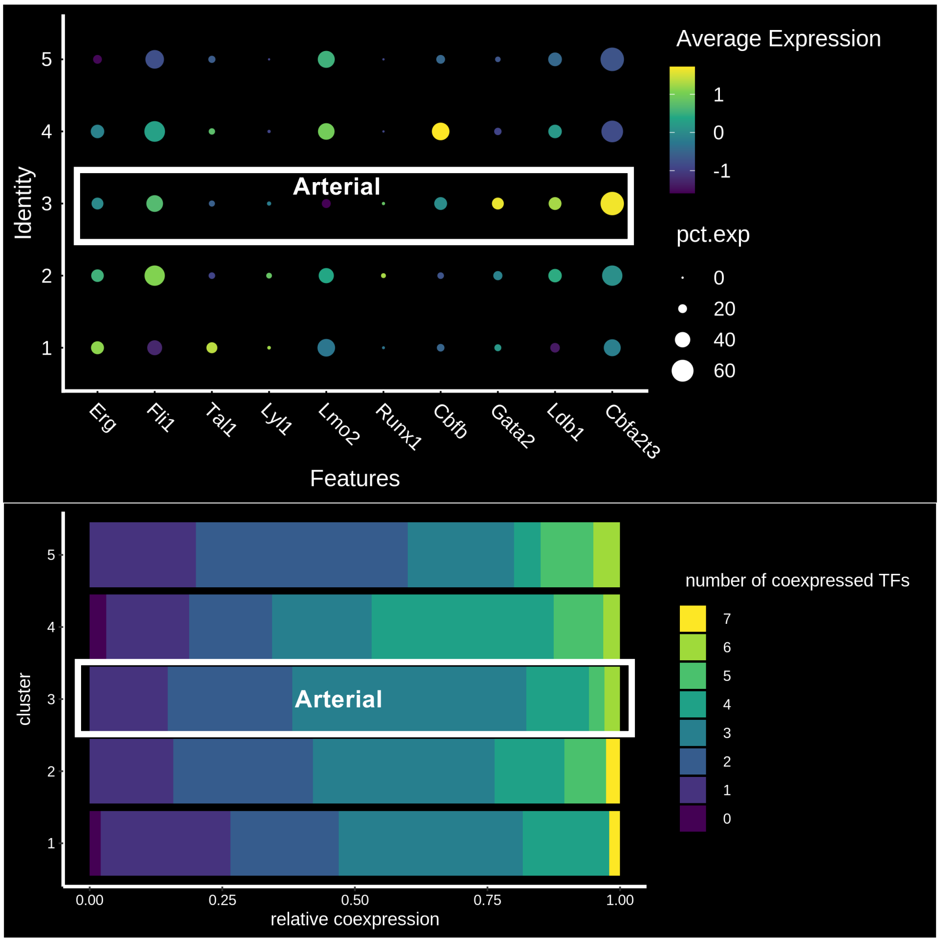

Supplementary Fig. 7

Lung EC

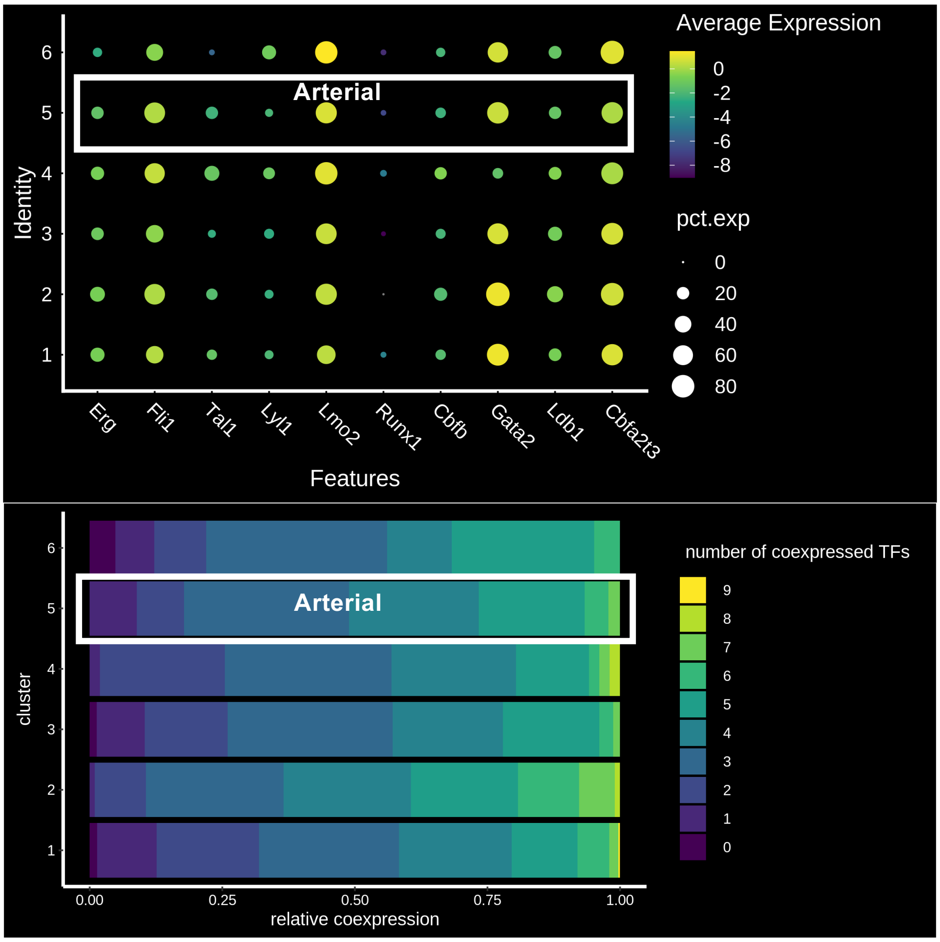

Pancreas EC

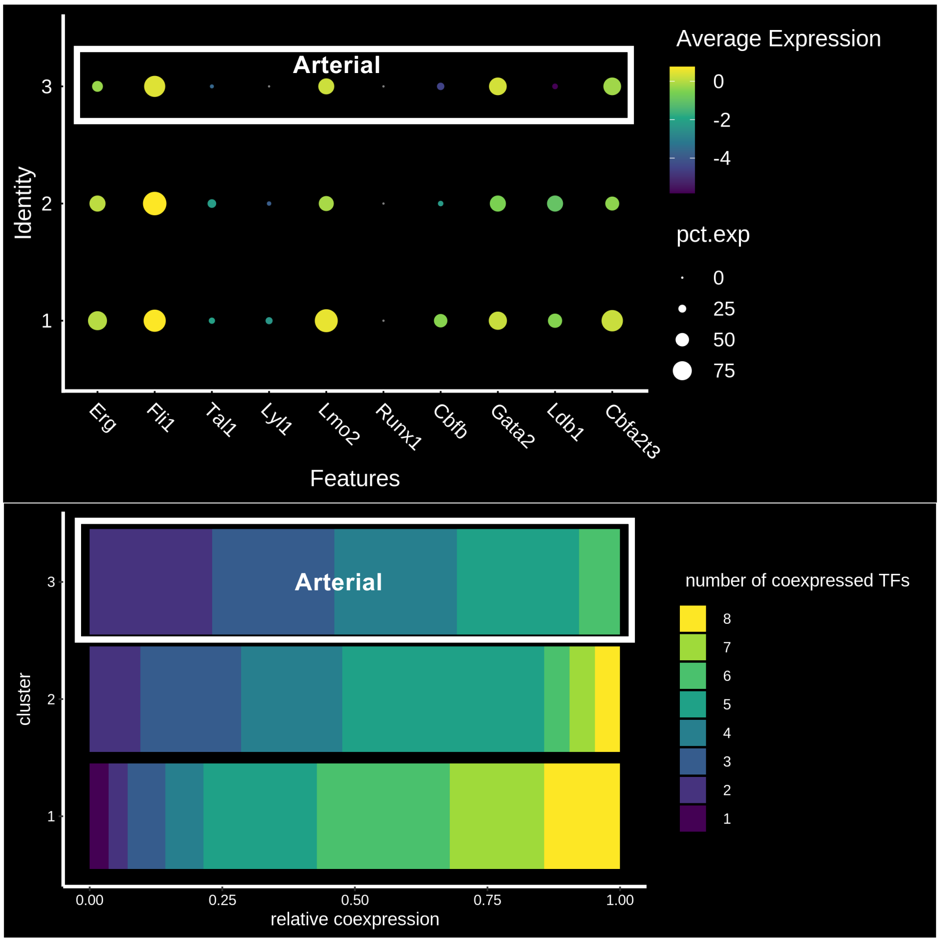

Supplementary Fig. 7: Analysis of transcription factor coding genes in endothelial cell clusters. For each tissue, the top panel shows a dot plot of the expression and percentage of expression of the indicated transcription factors in the endothelial cell clusters; the bottom panel shows the analysis of the co-expression between the transcription factors. The rectangles highlight arterial endothelial cell clusters.

a

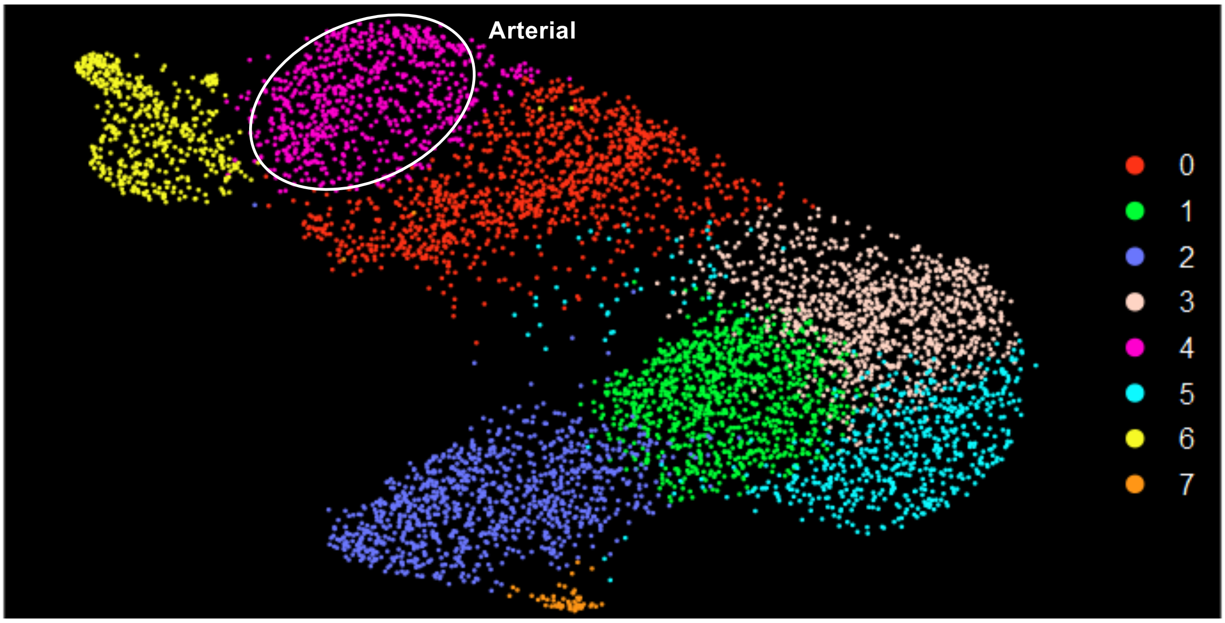
Arterial
0
1
2
3
4
5
6
7

b

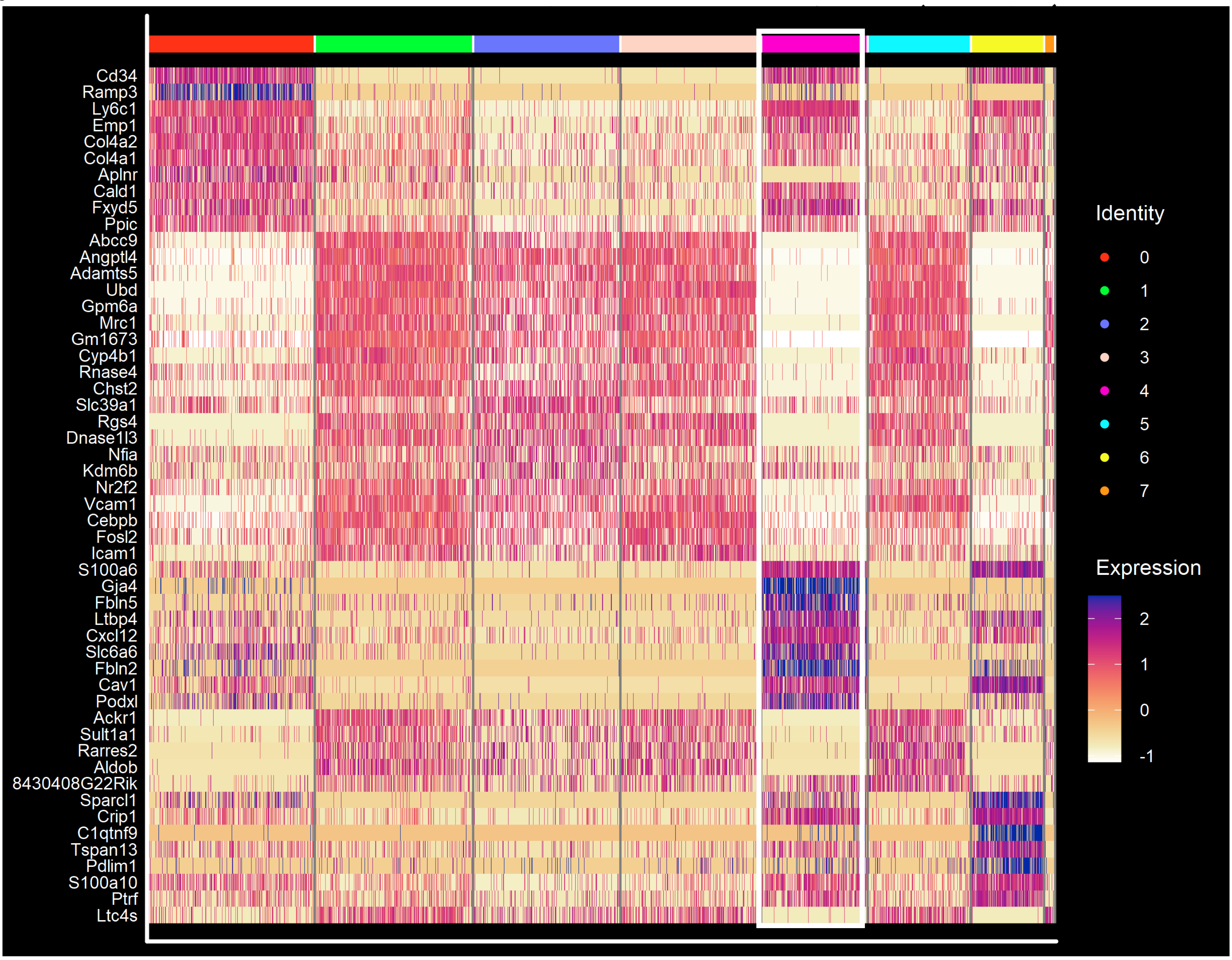
Cd34
Ramp3
Ly6c1
Emp1
Col4a2
Col4a1
Aplnr
Cald1
Fxyd5
Ppic
Abcc9
Angptl4
Adamts5
Ubd
Gpm6a
Mrc1
Gm1673
Cyp4b1
Rnase4
Chst2
Slc39a1
Rgs4
Dnase1l3
Nfia
Kdm6b
Nr2f2
Vcam1
Cebpb
Fosl2
Icam1
S100a6
Gja4
Fbln5
Ltbp4
Cxcl12
Slc6a6
Fbln2
Cav1
Podxl
Ackr1
Sult1a1
Rarres2
Aldob
8430408G22Rik
Sparcl1
Crip1
C1qtnf9
Tspan13
Pdlim1
S100a10
Ptrf
Ltc4s
Identity
0
1
2
3
4
5
6
7
Expression
2
1
0
-1

Supplementary Fig. 8 Results of the scRNA-seq clustering analysis of the bone marrow endothelial cells. a) UMAP plot highlighting the endothelial cell ; b) Heatmap of marker genes for the indicated cell clusters.

a

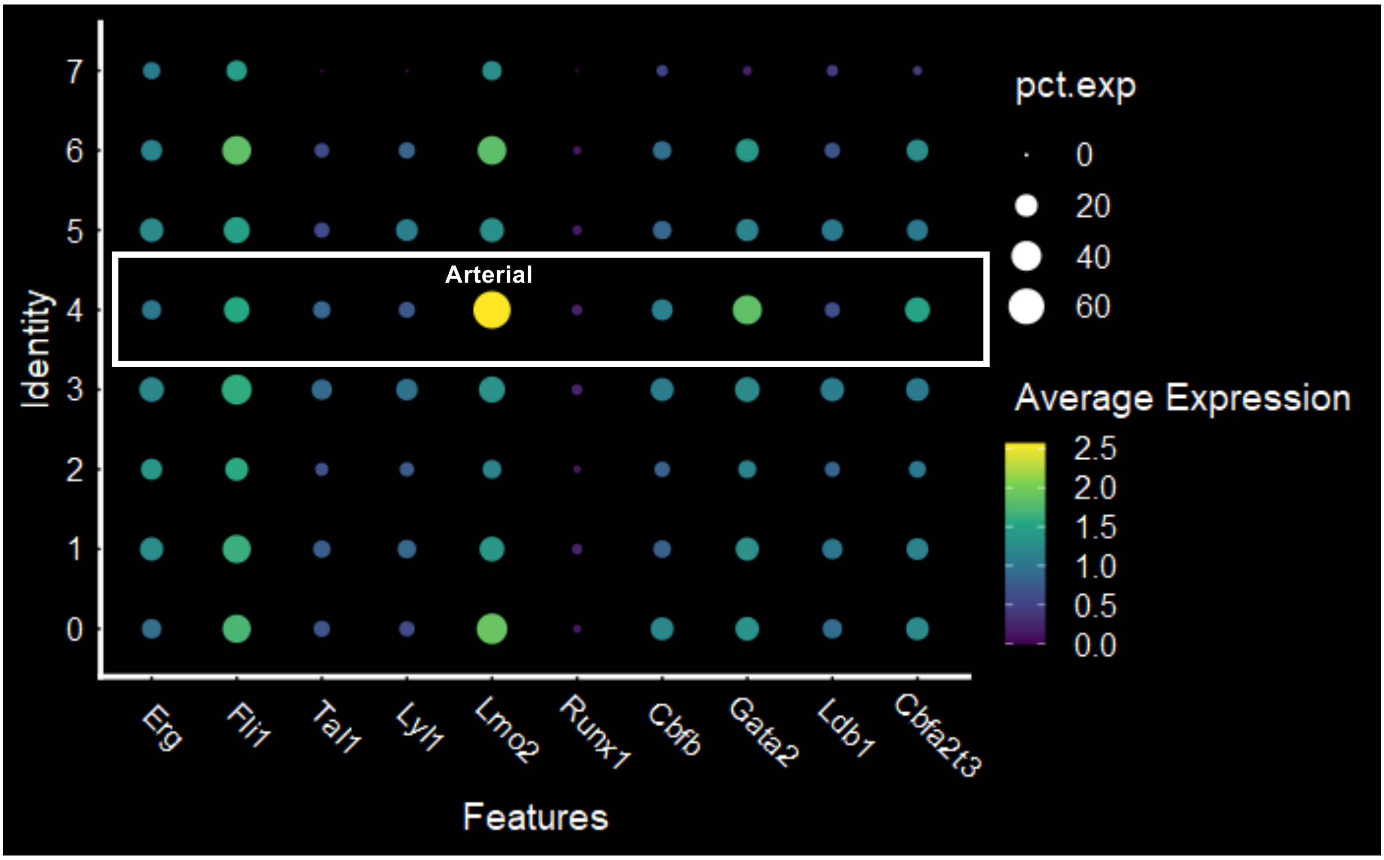
Arterial
pct.exp
0
20
40
60
Average Expression
2.5
2.0
1.5
1.0
0.5
0.0
Identity
7
6
5
4
3
2
1
0
Erg
Fli1
Tal1
Lyl1
Lmo2
Runx1
Cbfb
Gata2
Ldb1
Cbfa2t3
Features

b

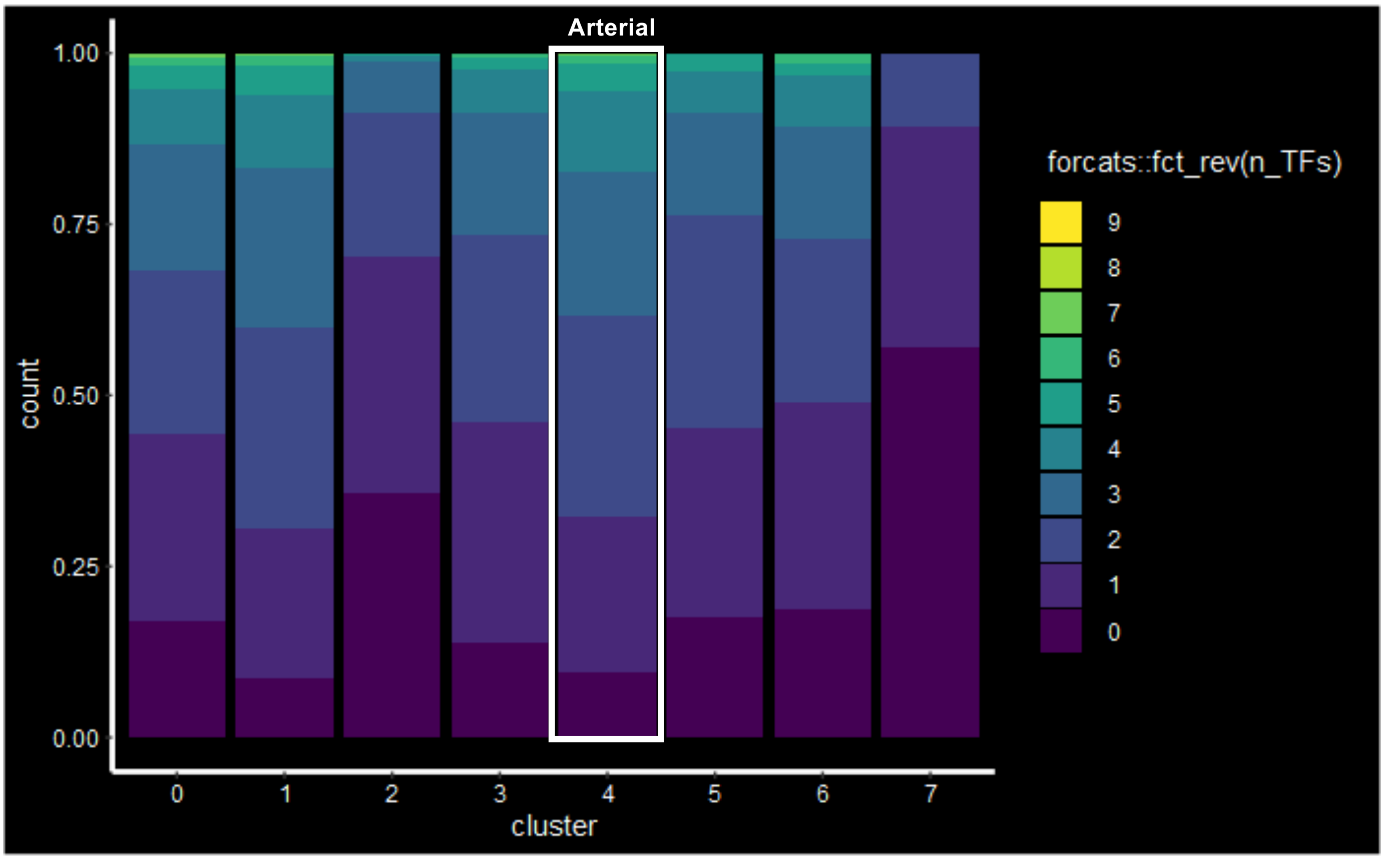
Arterial
count
1.00
0.75
0.50
0.25
0.00
0
1
2
3
4
5
6
7
cluster
forcats::fct_rev(n_TFs)
9
8
7
6
5
4
3
2
1
0

Supplementary Fig. 9: Analysis of transcription factor gene expression in the bone marrow endothelial cell clusters. a) Dot plot showing the expression and percentage of expression of the indicated transcription factors in the endothelial cell clusters; **b)** Analysis of the co-expression between the transcription factors.

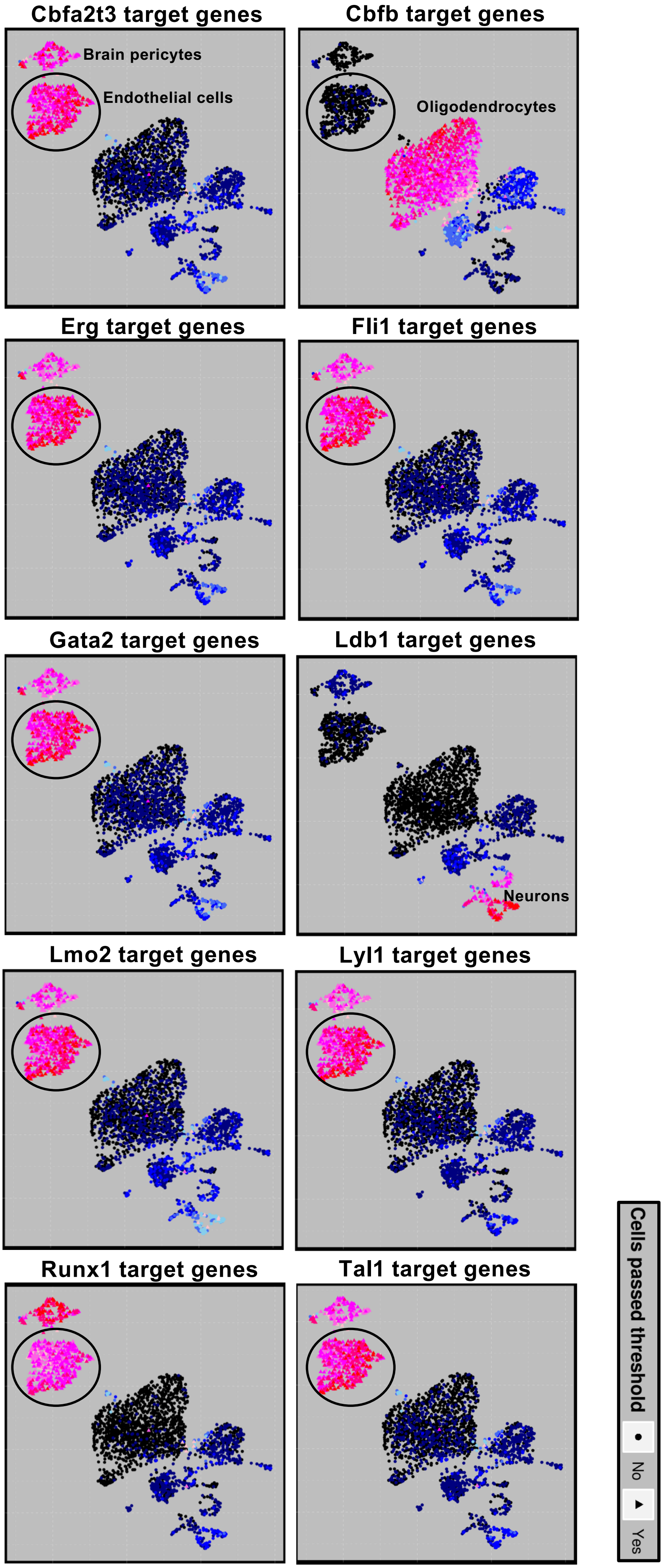
Cbfa2t3 target genes
Brain pericytes
Endothelial cells
Cbfb target genes
Oligodendrocytes
Erg target genes
Fli1 target genes
Gata2 target genes
Ldb1 target genes
Neurons
Lmo2 target genes
Lyl1 target genes
Runx1 target genes
Tal1 target genes
Cells passed threshold
No
Yes

Supplementary Fig. 10: UMAP plot highlighting the clusters in which the target genes of a given seed TFs are expressed. Only positive relationships are highlighted. The ellipse shows the endothelial cell cluster. Colors represent AUC value – cells that passed AUC threshold colored in pink, others in blue.

Supplementary Fig. 11

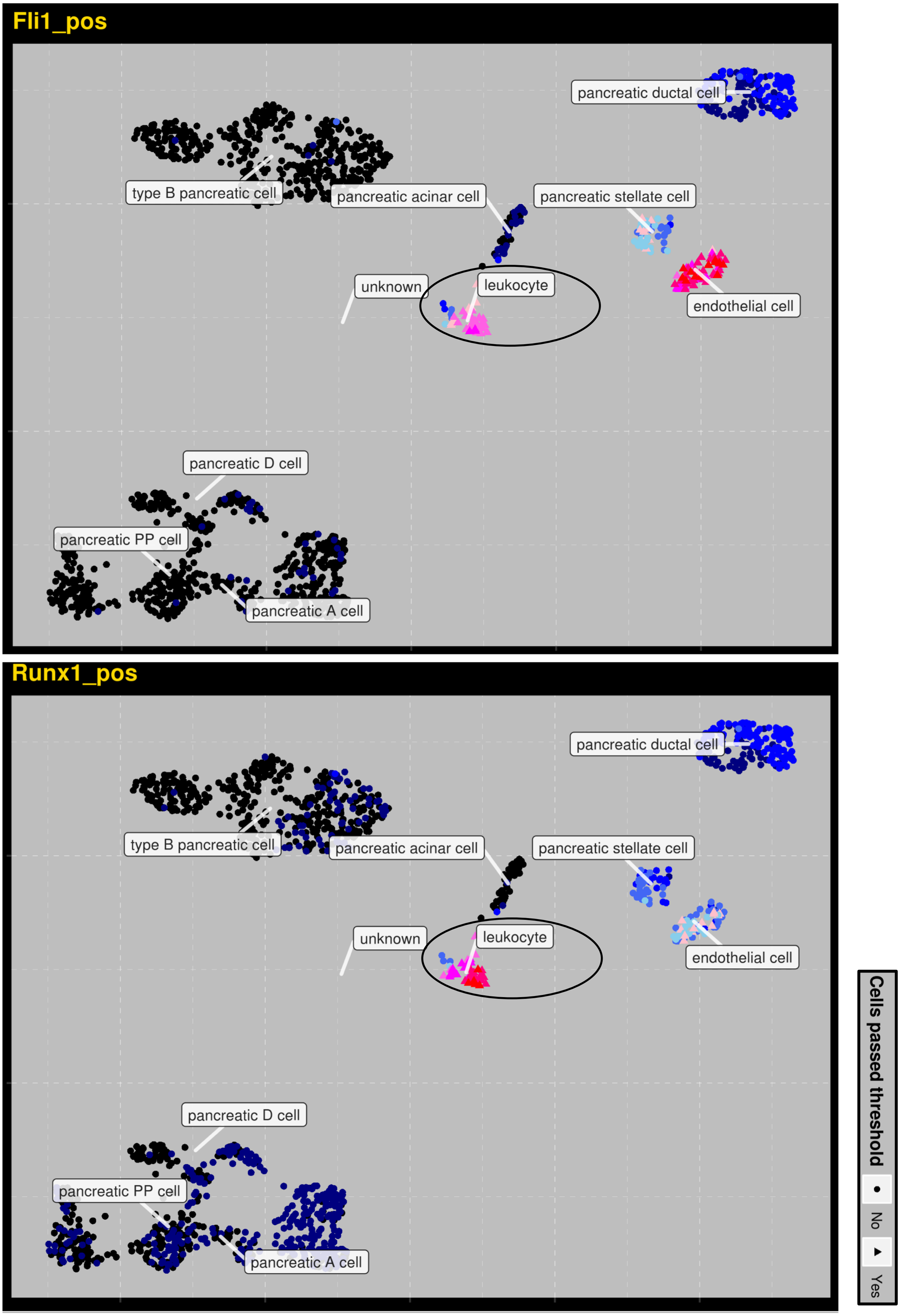

Supplementary Fig. 11: Overlap of genes between Fli1 and Runx1 only in leukocytes cluster. UMAP plot highlighting the clusters in which the target genes of a given seed TFs are expressed. Only positive relationships are highlighted. The ellipse shows the leukocytes cell cluster. Colors represent AUC value – cells that passed AUC threshold colored in pink, others in blue.

	c1	c2	c3	c4	c5	c6	c7	c8	c9	c10	c11	c12	c13	c14
Embryo_dataset1	0	22	20	30	1	8	0	97	0	0	11	0	0	100
Embryo_dataset2	3	5	5	2	5	1	5	2	20	9	3	6	4	0
Embryo_dataset3	0	71	75	44	0	87	0	1	8	0	84	3	0	0
Aorta	5	0	0	1	3	0	0	0	1	5	0	0	0	0
Brain	19	0	0	3	8	1	3	0	0	8	0	14	0	0
Diaphragm	3	0	0	0	1	0	0	0	2	1	0	3	0	0
Fat	7	1	0	4	21	1	64	0	27	23	1	31	0	0
Heart	23	1	0	4	45	1	18	0	3	15	1	15	95	0
Kidney	4	0	0	0	2	1	1	0	1	2	0	9	0	0
Limb_muscle	4	0	0	1	3	0	2	0	2	4	0	5	0	0
Liver	7	0	0	0	1	0	0	0	0	1	0	3	0	0
Lung	19	0	0	10	7	0	3	0	31	19	0	5	1	0
Mammary_gland	1	0	0	0	1	0	1	0	1	4	0	1	0	0
Pancreas	2	0	0	0	1	0	1	0	0	4	0	2	0	0
Trachea	3	0	0	1	1	0	2	0	4	5	0	3	0	0

Supplementary Table 1: Composition of the clusters from figure 2. The values represent percentage.

a

GO.ID	Term	p-value (FDR)
GO:0044703	Multi-organism reproductive process	0.0053
GO:0040007	Growth	0.0074
GO:0006457	Protein folding	0.0103
GO:0010033	Response to organic substance	0.0181
GO:0033043	Regulation of organelle organisation	0.0185

b

GO.ID	Term	p-value (FDR)
GO:0071496	Cellular response to external stimulus	0.0027
GO:0009314	Response to radiation	0.0098
GO:0043405	Regulation of MAP kinase activity	0.0102
GO:0006469	Negative regulation of protein kinase activity	0.0138
GO:0071248	Cellular response to metal ion	0.0209

Supplementary Table 2: Gene ontology analysis of adult and embryonic cell clusters from figure 2.
a) List of the top 5 GO term of genes most differentially expressed in the embryonic cell clusters.
b) List of the top 5 GO term genes most differentially expressed in the adult cell clusters.

Gene symbol	Number of common tissues	Name of tissues
Cd63	5	fat, kidney, limb_muscle, lung, trachea
Gsn	5	fat, heart, kidney, limb_muscle, trachea
Mt1	5	diaphragm, fat, kidney, limb_muscle, trachea
Myl9	5	brain, diaphragm, fat, limb_muscle, trachea
Rgs5	5	aorta, brain, diaphragm, limb_muscle, trachea
Acta2	4	diaphragm, fat, limb_muscle, trachea
Art3	4	brain, diaphragm, kidney, mammary_gland
Bgn	4	brain, kidney, limb_muscle, trachea
Cd44	4	diaphragm, kidney, limb_muscle, mammary_gland
Ndrg2	4	aorta, diaphragm, limb_muscle, trachea
Notch2	4	aorta, diaphragm, kidney, limb_muscle
Pex7	4	aorta, kidney, limb_muscle, lung
Stip1	4	aorta, fat, kidney, mammary_gland

Supplementary Table 3: Identification of marker genes in $Runx1^{+}$ adult endothelial populations.
